# An unusual growth in the nail matrix: A case of superficial acral fibromyxoma

**DOI:** 10.1002/ski2.121

**Published:** 2022-05-03

**Authors:** Genevieve Ho, Caroline Kurek, David Stewart, Linda K. Martin

**Affiliations:** ^1^ Melanoma Institute Australia Sydney New South Wales Australia; ^2^ Faculty of Medicine and Health University of Sydney Sydney New South Wales Australia; ^3^ Histopathology Douglass Hanly Moir Pathology Sydney New South Wales Australia; ^4^ Royal North Shore Hospital Sydney New South Wales Australia; ^5^ Faculty of Medicine University of New South Wales Sydney New South Wales Australia

## Abstract

We describe a case of superficial acral fibromyxoma arising within the germinal matrix of the index finger. This is an uncommon localisation of this relatively newly described benign soft tissue tumour. Herein, we discuss the varied clinical presentation, distinguishing histopathological features and important differential diagnoses for this condition.

## Graphical abstract

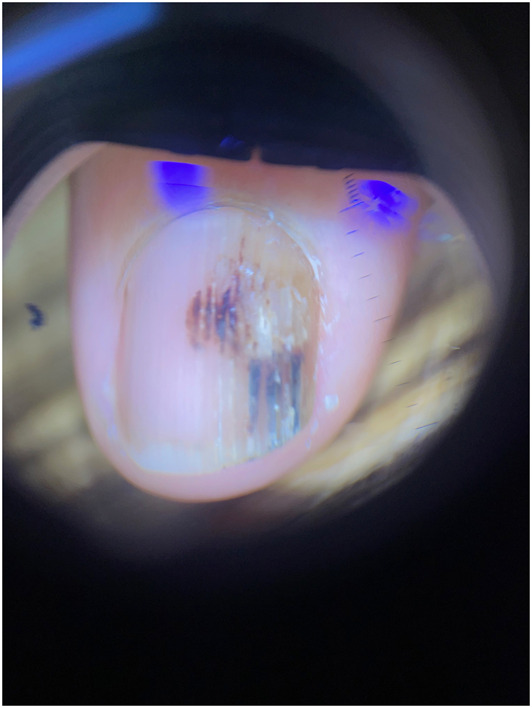

1

**What's already known about this topic?**

- SAF is a relatively new soft tissue tumours described to affect periungual skin. It has a host of differentials including benign and malignant tumours, and is distinguished by its histological and immunohistochemistry features (strong positive for CD34 and CD99).

**What does this study add?**

- This case describes the clinical presentation and pathological features of a case of SAF affecting the germinal matrix which has rarely been reported.

## 2 INTRODUCTION

Superficial acral fibromyxoma (SAF), also known as digital fibromyxoma, is a rare benign soft tissue tumour with a predilection for the digits, first described in 2001 in a series of 31 cases.^1^ As of 2019, there have been 314 cases described in the literature, and affect the fingers and toes in similar proportions.^2^ We present a unique case of this relatively newly described tumour arising within the germinal nail matrix, a location that has not been commonly described.

**TABLE 1:** Main clinical tumoural differential diagnoses of subungual tumours

Clinical differential diagnoses	
Benign	Malignant
Acquired digital fibrokeratoma (ungual variants)	Subungual melanoma
Glomus tumour	Squamous cell carcinoma
Subungual keratoacanthoma	
Subungual exocytosis	
Superficial acral fibromyxoma	
Onychomatricoma	
Onychopapilloma	

## 3 CASE REPORT

**FIGURE 1:**
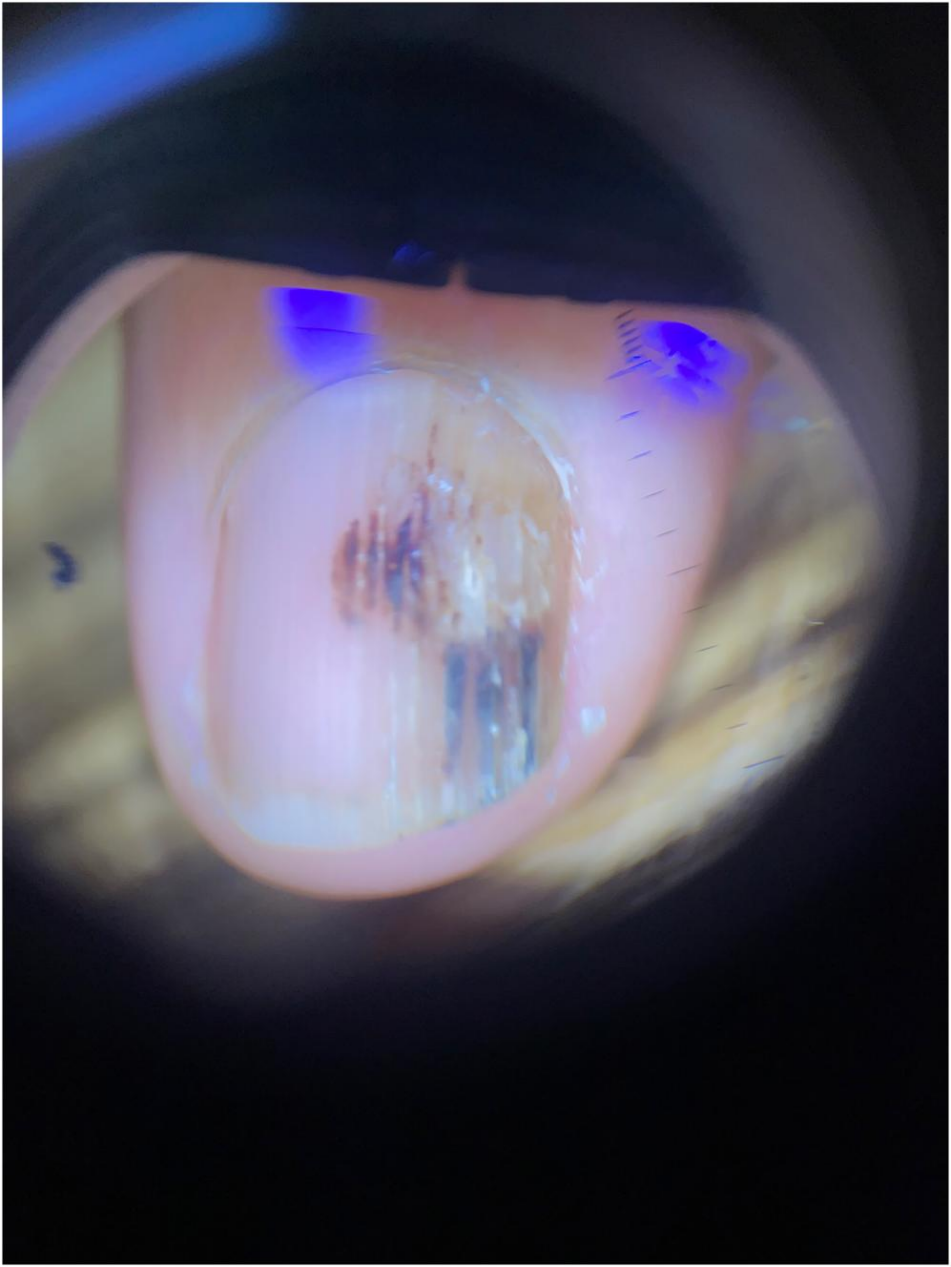
Dermoscopy image of patient's right index fingernail showing focal longitudinal trachyonychia, onychorrhexis and subungual haemorrhage

A 48‐year‐old woman presented with a 3‐month history of changing pigmentation on the nail of her right index finger. She reported a history of a narrow band of longitudinal melanonychia present for at least 5–6 years prior, which became more noticeable as the colour darkened and widened. She described occasional discomfort at the base of the nail but did not recall any history of trauma. She has frequent manicures, and works as an artist with regular contact with wet surfaces, fibres and soils. The patient had a history of a benign breast lump and benign ovarian mass; but was otherwise healthy. She was of mixed South American and European ancestry and had Fitzpatrick phototype IV skin. She had no personal history of melanoma but a family history of melanoma in a second degree relative. On examination, there was localised nail dystrophy with trachyonychia and subungual haemorrhage. Hutchinson sign was negative (Figure 1).

**FIGURE 2:**
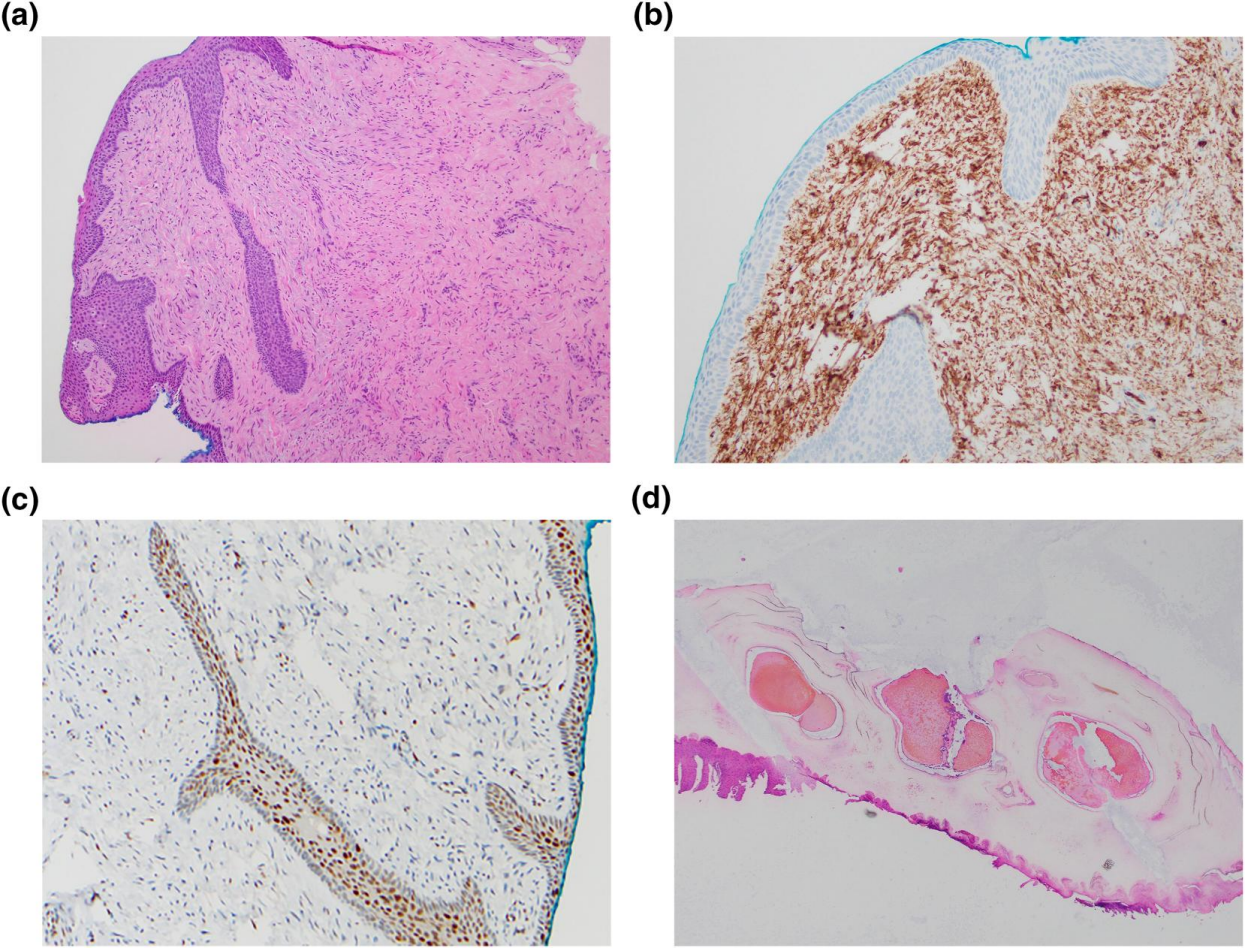
(a) Bland spindle and stellate cells with a myxoid stroma within the superficial dermis in a loosely fascicular pattern (H&E, 100x magnification); (b) Tumour cells are strong and diffusely positive for CD34 (200x magnification); (c) Loss of RB‐1 expression in the lesional stroma, with only uptake highlighting blood vessels, mast cells and lymphocytes (200x magnification); (d) Low power magnification showing intracorneal haemorrhage (H&E stain, 40x magnification)

Due to the history of previous melanonychia, progressive nail dystrophy and recent rapid change, the patient underwent nail matrix biopsy to rule out melanoma. A pedunculated lesion in the germinal matrix was evident, which was excised and sent for histology. Histopathology showed a polypoid tumour based within the superficial dermis, composed of bland spindle and stellate cells within a myxoid and collagenous stroma (Figure 2a). The cells had a loosely fascicular pattern of growth and mild variation in nuclear size. There were prominent interspersed vessels and occasional mast cells. Mitoses were inconspicuous, and there was no necrosis, pleomorphism or perineural permeation. Strands of epithelium and cystic cavities noted within the lesion had no evidence of trichilemmal keratinisation on deep levels. Immunohistochemistry showed diffuse and strong positivity for CD34 and CD99 in the lesional stroma (Figure 2b). Immunostains were negative for epithelial membrane antigen (EMA), smooth muscle actin, Sox10, desmin and showed loss of RB‐1 expression (Figure 2c). This was consistent with a diagnosis of SAF. Histopathology of the nail plate also demonstrated intracorneal haemorrhage (Figure 2d), which was unusual given the lack of injury historically. The lesion was completely excised during the biopsy, and the patient did not experience further recurrence.

## 4 DISCUSSION

Superficial acral fibromyxoma is an emerging differential diagnosis for benign soft tissue tumours affecting the digits. Although incidence data is lacking, it has been increasingly described and may be more common initially assumed. Clinically, up to 97% of finger tumours grow in close proximity to the nail apparatus, and subungual lesions have been reported in 43% of finger cases, others being periungual.^3^ It usually presents as a slow growing and asymptomatic pink to flesh coloured nodule often leading to a delay in presentation, with 36% reported to erode into bone.^4^ Tumours arising within the germinal matrix have been rarely reported. Sub‐matricial SAF has been described to cause the clinical appearance of pseudo‐clubbing, onychogryphosis and triangular macroluna (large ‘half‐moons’).^5^ Another case involving the lateral nail fold comprised a lesion extending below the nail matrix, presenting as just onychoschizia of the nail over the area of the mass.^6^

The description of longitudinal melanonychia prior to onset of the mass is unusual and warranted concerns of a malignant differential diagnosis. This however was most likely explained by haemorrhage rather than true melanin deposition. Superficial acral fibromyxoma associated with haemorrhage has previously been reported to mimic a pigmented differential.^7^ Our patient observed the most change when she started more occupation related handling of wet materials, such as soils, which may have accentuated the presentation. There are however no known risk factors to development of SAF.

Histologically, the tumour is characterised by stellate, ovoid or spindle shaped cells, in a storiform, lobular or fascicular growth pattern. The stroma is myxoid or myxocollagenous and the tumour is non‐encapsulated. Margins may be irregular, and some may extend into subcutaneous tissue, underlying fascia or even bone.^3^ Immunohistochemistry is positive for CD34 and CD99, more variably for EMA, and are always negative for S100, distinguishing it from neurofibroma, which is the most common pathological differential diagnosis.^3^, ^4^ Retinoblastoma‐1 (RB‐1) is a tumour‐suppressor gene, which is negative in SAF. It's role in the diagnosis of SAF and other “RB1‐deleted soft tissue tumours” has been more recently described.^8^

Clinical differential diagnoses of subungual tumours presenting with nail dystrophy include the benign glomus tumours, subungual keratoacanthoma, subungual exocytosis, digital mucous cysts, onychomatricoma and onychopapillloma^4^, ^9^; and the malignant melanoma and squamous cell carcinoma (Table 1).^4^ The main histological differentials include acquired digital/ungual fibrokeratoma (clinically has a classically hyperkeratotic collarete at its base) and periungual fibroma (usually periungual), both of which have less cellularity than SAF, and do not extend as deeply into the dermis and subcutis as SAF.^1^, ^10^ Other differentials to consider include.

Neurofibroma (distinguished by S100 positivity), dermatofibrosarcoma protuberans of myxoid type (prefers trunks and extremities and EMA negative), superficial angiomyxoma (usually head and neck), low‐grade fibromyxoid sarcoma and myxofibrosarcoma (rare on acral surfaces and negative for CD34). These should be distinguished by clinical, histological and immunohistochemistry features.^4^

Surgical excision is the recommended management, however pre‐operative imaging with radiological assessment (X‐Ray, Ultrasound, Magnetic resonance imaging) may be required to assess for bony involvement.^3^

There are reports of recurrence in 20%–25% of cases, although no reports of malignant transformation are noted to date.^4^ Our case highlights this benign tumour as an emerging differential for benign and malignant tumours of the nail unit.

## CONFLICT OF INTEREST

The authors have no conflict of interest to declare.

## AUTHOR CONTRIBUTION

**Genevieve Ho:** Conceptualization (lead); data curation (lead); writing – original draft (lead); writing – review & editing (lead). **Caroline Kurek:** Data curation (equal); Investigation (equal); Writing – review & editing (equal). **David Stewart:** Conceptualization (equal); data curation (equal); Writing – review & editing (equal). **Linda K Martin:** Data curation (equal); Supervision (lead); Writing – review & editing (equal).

## ETHICS STATEMENT

The patient has consented for use of de‐identified clinical images and information in this report; ethics review was not required as it is a retrospective case report.

## Data Availability

Data sharing is not applicable to this article as no new data were created or analyzed in this study.
